# Phenotypic, proteomic, and functional analyses of cytokine-induced memory-like NK cells show two distinct subsets based on CD16 expression

**DOI:** 10.1038/s41598-025-20947-1

**Published:** 2025-10-23

**Authors:** Sofía Carreira-Santos, Marina González-Sánchez, Nelson López-Sejas, Fakhri Hassouneh, Lauro González-Fernández, Inmaculada Jorge, Esther Durán, Alejandra Pera, Jesús Vázquez, Rafael Solana, Raquel Tarazona, Javier G. Casado

**Affiliations:** 1 Immunology Unit, Department of Physiology, Universidad de Extremadura, Cáceres, Spain; 2 Department of Cell Biology, Physiology and Immunology, Universidad de Córdoba, Córdoba, Spain; 3 Immunology and Allergy Group (GC01), Instituto Maimónides de Investigación Biomédica (IMIBIC), Córdoba, Spain; 4 Departamento de Bioquímica y Biología Molecular y Genética, Grupo de Investigación Señalización Intracelular y Tecnología de la Reproducción (SINTREP), Instituto de Investigación INBIO G+C, Universidad de Extremadura, Cáceres, Spain; 5 Cardiovascular Proteomics Laboratory, Centro Nacional de Investigaciones Cardiovasculares Carlos III (CNIC), Madrid, Spain; 6 Centro de Investigación Biomédica en Red, Enfermedades Cardiovasculares (CIBERCV), Madrid, Spain; 7 Anatomy and Comparative Pathological Anatomy Unit, Department of Animal Medicine, Faculty of Veterinary Medicine, Universidad de Extremadura, Cáceres, Spain; 8 Immunology and Allergy Service, Reina Sofia University Hospital, Cordoba, Spain; 9 Institute of Molecular Pathology Biomarkers, Universidad de Extremadura, Cáceres, Spain; 10 RICORS-TERAV Network, Instituto de Salud Carlos III (ISCIII), Madrid, Spain

**Keywords:** NK cells, Cytokine-induced memory-like NK cells, CD16, Degranulation capacity, Proteomics, Enrichment analysis, Cancer immunotherapy, Cancer, Immunology

## Abstract

NK cells are innate lymphoid cells that can acquire a memory-like phenotype in vitro when stimulated with IL-12, IL-15, and IL-18. These cytokine-induced memory-like (CIML) NK cells exhibit prolonged lifespan and increased cytotoxicity, making them ideal for immunotherapy. This study characterizes two CIML NK cell subsets based on CD16 expression. NK cells were isolated from the peripheral blood of healthy donors and stimulated overnight to induce a memory-like phenotype. After seven days, we analyzed the phenotype and degranulation potential of CD16−/CD56 + and CD16+/CD56 + cells. The subsets were purified by fluorescence-activated cell sorting (FACS) and examined using high-throughput multiplexed quantitative proteomics. CD16 − cells showed higher levels of activating receptors, increased Granulysin expression, and lower inhibitory receptor expression compared to CD16 + cells. Functionally, CD16 − cells exhibited greater degranulation capacity, as determined by CD107a/b expression, when co-incubated with K562 and melanoma cells. Proteomic profiling identified 35 differentially expressed proteins out of 4,750, with 22 downregulated and 13 upregulated in the CD16 − subset. Key proteins included Granzyme family proteins, NCAM1, CALM1, CD247, and Fc receptors. This study provides a detailed characterization of CIML NK cells based on CD16 expression. Our findings highlight the molecular and functional diversity of CIML NK cells and may guide improved cancer immunotherapy strategies.

## Introduction

Natural Killer (NK) cells are cytotoxic lymphocytes that play a key role within the innate immune system, capable of recognizing and eliminating virus-infected cells and tumor cells^1,2^. Their cytolytic function is tightly regulated through a balance of signals from various inhibitory (e.g., KIRs, TIGIT) and activating receptors (e.g., NKG2D, CD16) on their surface, allowing them to distinguish and selectively target abnormal or stressed cells while sparing healthy tissue^2,3^.

Human NK cells from peripheral blood can be classified based on the expression of two surface markers: CD56 (NCAM-1) and CD16a (FcγRIIIa)^4,5^. The CD16− CD56bright subset is characterized by lower cytotoxicity but has a greater ability to secrete cytokines, like interferon gamma (IFN-γ), upon activation^6^. In contrast, the CD16+ CD56dim subset is highly cytotoxic, using granzyme (e.g., GZMA, GZMB) and perforin release or death receptor-mediated pathways (e.g., FASL, TRAIL) to eliminate target cells^5–7^. Additionally, CD16 plays a key role in antibody-dependent cellular cytotoxicity (ADCC) against opsonized cells, which is clinically relevant in tumor-targeting responses^8,9^.

Recent studies have demonstrated that under specific conditions, NK cells can acquire a memory-like phenotype, marked by clonal expansion, rapid degranulation, and increased cytokine production upon reactivation^10–13^. Among these, cytokine-induced memory-like (CIML) NK cells, generated through brief stimulation with IL-12, IL-15, and IL-18, exhibit enhanced functional and phenotypic characteristics, such as enhanced cytotoxicity and extended survival in immunosuppressive environments, making them promising candidates for cancer immunotherapy^14–18^. Preclinical and clinical studies have highlighted their potential in treating various cancers, such as ovarian cancer^6^, melanoma^19^, acute myeloid leukemia (AML)^20^, and lymphoma^19^.

In a previous work, we characterized the enhanced phenotype and functional capacity of CIML NK cells after in vitro expansion, noting increased expression of activating and inhibitory receptors, enhanced degranulation capacity against target cells, and higher production of IFN-γ and GZMB compared to unstimulated NK cells^21^. However, there remains a significant gap in understanding the proteomic expression pattern in CIML NK cell subsets, particularly the CD16 − and CD16 + populations, which may underline their distinct functional properties.

In this study, we aim to systematically compare the phenotype and functionality of CD16− and CD16+ CIML NK cell subsets after a 7-day culture period. Using fluorescence-activated cell sorting (FACS) to isolate these populations, we performed proteomics analysis to identify potential protein-level differences in the protein profile. Our findings revealed distinct expression patterns on activating and inhibitory receptors between the subsets, as well as variations in cytotoxic protein levels and degranulation responses to target cells like the K562 cell line and melanoma cell lines.

Proteomic profile revealed 35 differentially expressed proteins between the subsets, linked to pathways involved in NK cell-mediated cytotoxicity such as FcγRIII, CAPG, and GZMK. These results highlight unique molecular features that may drive functional specialization of CD16− and CD16+ CIML NK cells. Therefore, our findings offer valuable insights into the molecular and functional diversity of CIML NK cells and may inform the development of more effective strategies for cancer immunotherapy.

## Results

### Two distinct subsets of CIML NK cells can be distinguished based on CD16 expression

**Fig. 1:**
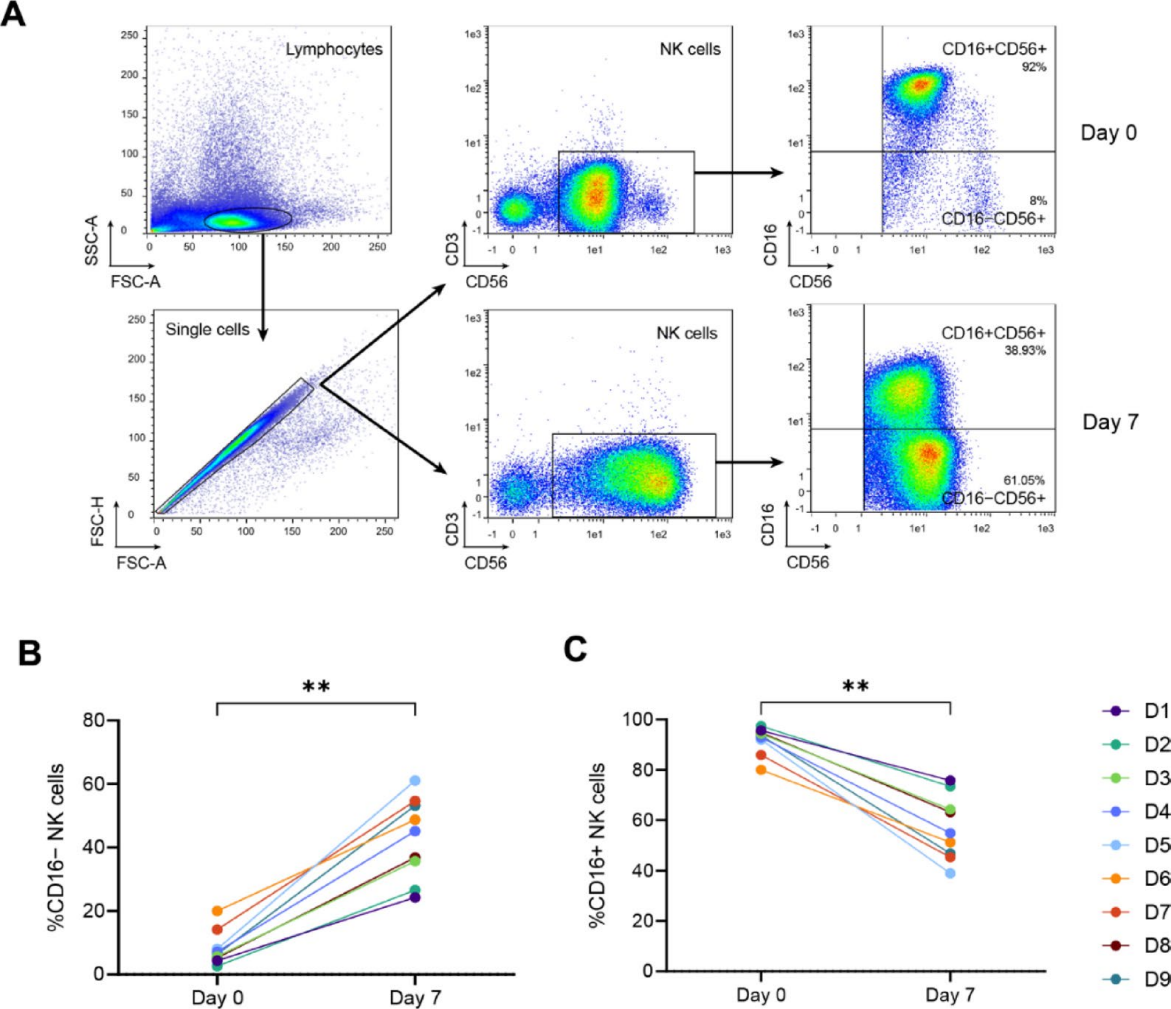
Gating strategy and evolution of NK cell subsets over time. (**A**) Lymphocytes were gated by size and granularity (FSC vs. SSC), and NK cells were identified within the CD56+ CD3− lymphocyte gate. NK cells were further subdivided into CD16− CD56+ and CD16+ CD56+ subsets, and their respective marker expression was analyzed. (**B**) Percentage of CD16− NK cells on Day 0 and Day 7 for all nine donors. (**C**) Percentage of CD16+ NK cells over the same period. Statistical significance was determined using the Wilcoxon signed-rank test, ** *p* ≤ 0.01.

The distribution of NK cells based on CD16 and CD56 expression was evaluated at baseline (Day 0) and after stimulation with IL-12/15/18, followed by a 7-day culture with IL-15 (Day 7). At Day 0, as expected, CD16+ CD56+ NK cells represented the major NK cell subset (Fig. 1A).

In all nine donors, the proportion of CD16− NK cells increased significantly between Day 0 and Day 7 (*p* = 0.004). The mean percentage of CD16− NK cells rose from 8.16% ±5.48% at baseline to 42.92% ±12.84% in CIML NK cells on Day 7 (Fig. 1B). Consequently, the percentage of CD16+ NK cells declined significantly over the same time period (*p* = 0.004), decreasing from 91.84% ±5.48% at baseline to 57.08% ±12.84% in CIML NK cells on Day 7 (Fig. 1C).

### Phenotypic profiles of CD16− and CD16+ CIML NK cells exhibit significant differences

After 7 days of culture, no significant differences were observed in the expression of CD25 and CD69 between CD16− and CD16+ CIML NK cells. The mean percentage of CD25 expression was 77.92% ±11.98% for CD16− and 74.25% ±24.22% for CD16+ CIML NK cells. CD69 expression was equally high, at 92.55% ±8.06% for CD16− and 92.70% ±7.49% for CD16+ CIML NK cells, indicating that both subsets were highly activated following IL-12/15/18 stimulation.

**Fig. 2:**
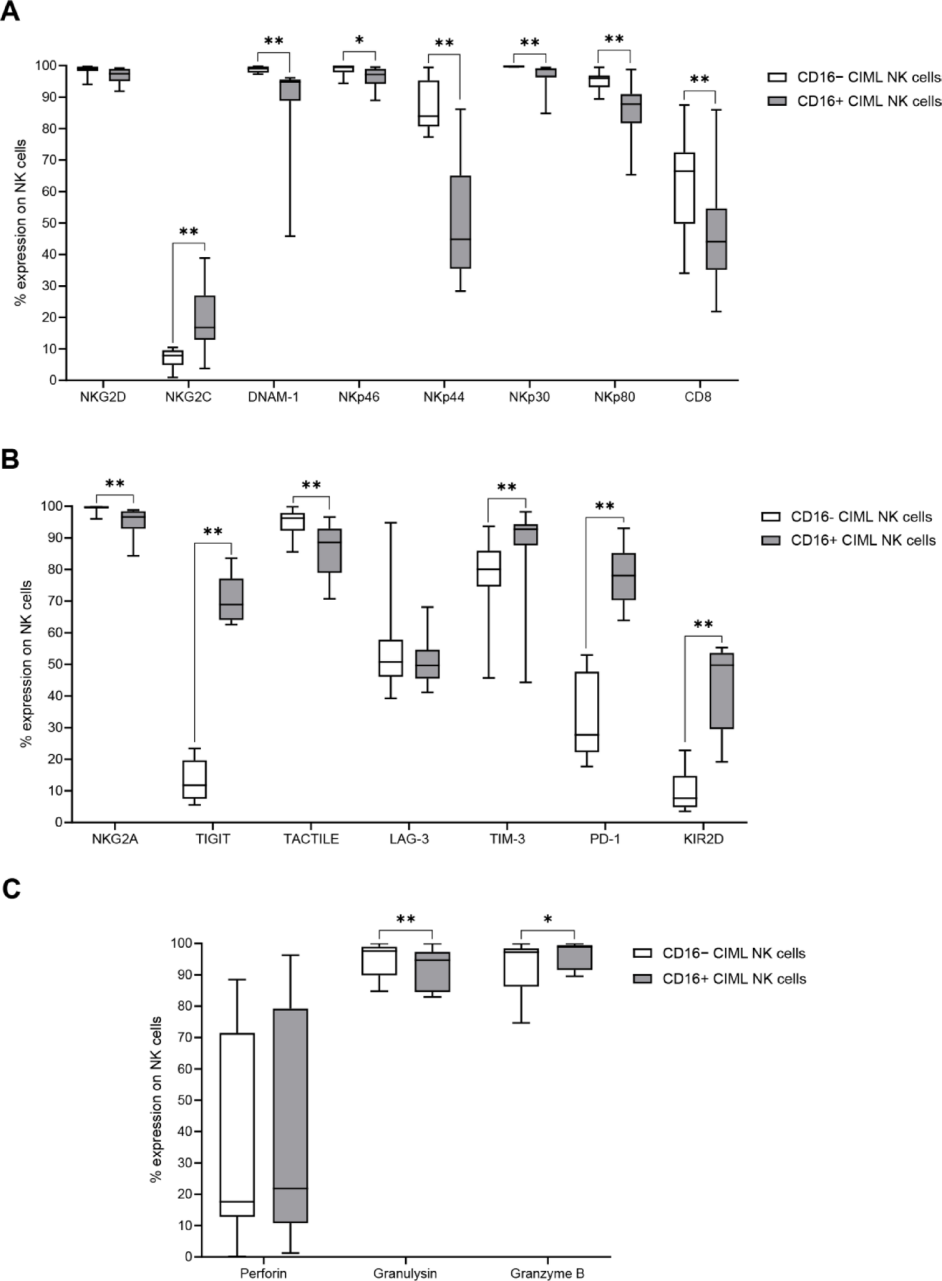
Phenotypic profiles of CD16− and CD16+ CIML NK cells after 7 days of culture. (**A**) Expression of activating receptors and CD8. (**B**) Expression of inhibitory receptors. (**C**) Cytotoxic protein expression. Statistical significance was assessed using the Friedman test with pairwise comparisons (Durbin-Conover test), * *p* ≤ 0.05, ** *p* ≤ 0.01.

In terms of activating receptor expression, CD16− CIML NK cells displayed significantly higher levels of DNAM-1, NKp46, NKp44, NKp30, NKp80, and CD8 compared to CD16+ cells (*p* = 0.004 for DNAM-1, NKp44, NKp30, and NKp80; *p* = 0.039 for NKp46; *p* = 0.008 for CD8). However, NKG2C expression was significantly lower in CD16− CIML NK cells (*p* = 0.004). No significant differences were observed for NKG2D expression (Fig. 2A).

Regarding inhibitory receptor expression, CD16− CIML NK cells showed higher levels of NKG2A and TACTILE (*p* = 0.004 for both) but lower levels of TIGIT, TIM-3, PD-1, and KIR2D compared to CD16+ cells (*p* = 0.004 for TIGIT, PD-1, and KIR2D; *p* = 0.008 for TIM-3) (Fig. 2B).

In terms of cytotoxic protein expression, CD16− CIML NK cells had significantly higher granulysin levels (*p* = 0.004), but lower granzyme B levels (*p* = 0.018) compared to CD16+ cells. No significant differences were observed in perforin expression (Fig. 2C), and median fluorescence intensity (MFI) values showed no differences across conditions (Supplementary Fig. 1).

### CD16− CIML NK cells show enhanced degranulation capacity compared to CD16+ cells

**Fig. 3:**
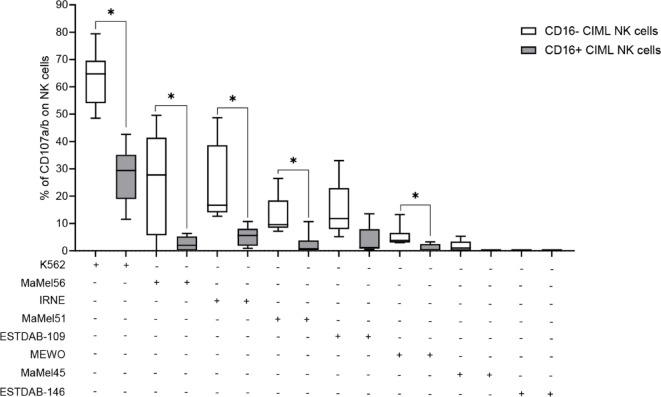
Degranulation capacity of CD16− and CD16+ CIML NK cells after 7 days of culture. Basal degranulation (in the absence of target cells) was subtracted to calculate specific degranulation capacity. Statistical significance was assessed using the Wilcoxon signed-rank test, * *p* ≤ 0.05.

To investigate the functional differences between CD16− and CD16+ CIML NK cells, we assessed their degranulation capacity by measuring CD107a/b expression during in vitro co-culture with target cell lines. CIML NK cells (effectors) were cultured with K562 or various melanoma cell lines (targets) at a 1:1 E: T ratio, and CD107a/b expression was analyzed by flow cytometry on gated CD16− CD56+ and CD16+ CD56+ cells (Fig. 3).

CD16− CIML NK cells exhibited greater degranulation activity against K562, MaMel56, IRNE, MaMel51, and MEWO cell lines compared to CD16+ cells (*p* = 0.020 for K562; *p* = 0.022 for MaMel56; *p* = 0.031 for IRNE, MaMel51, and MEWO). In contrast, MaMel45 and ESTDAB-146 elicited minimal degranulation in both CIML NK cell subsets.

Since NK cell interactions with target cells are known to induce CD16 downregulation, we cannot exclude that a percentage of CD16+ CIML NK cells may have downregulated CD16 expression during co-incubation with K562 and melanoma cell lines in the CD107a/b degranulation assay. In support of this, here we observed a significant downregulation of CD16 expression in CIML NK cells co-incubated with K562 cells, compared to control (spontaneous degranulation) CIML NK cells (% of CD16 expression: 24.70% ±9.83% and 40.70% ±6.97% respectively; *p* = 0.004). Moreover, no significant differences in CD16 expression were found when comparing CIML NK cells co-incubated with melanoma cell lines and control CIML NK cells (Supplementary Fig. 4).

### Differences in the proteomic profile of CD16− and CD16+ CIML NK cells reveal distinct immune signatures

Proteomic analysis was performed on sorted CD16− and CD16+ CIML NK cells to identify differences at protein expression level between these subsets. Protein extracts from four donors were analyzed using a multiplexed quantitative proteomic approach. A total of 4,750 proteins were identified, and 44.75% of them were classified within the “Innate Immune System” category (R-HSA-168249), as per the Reactome Knowledge Database (https://reactome.org)^22^.

**Fig. 4:**
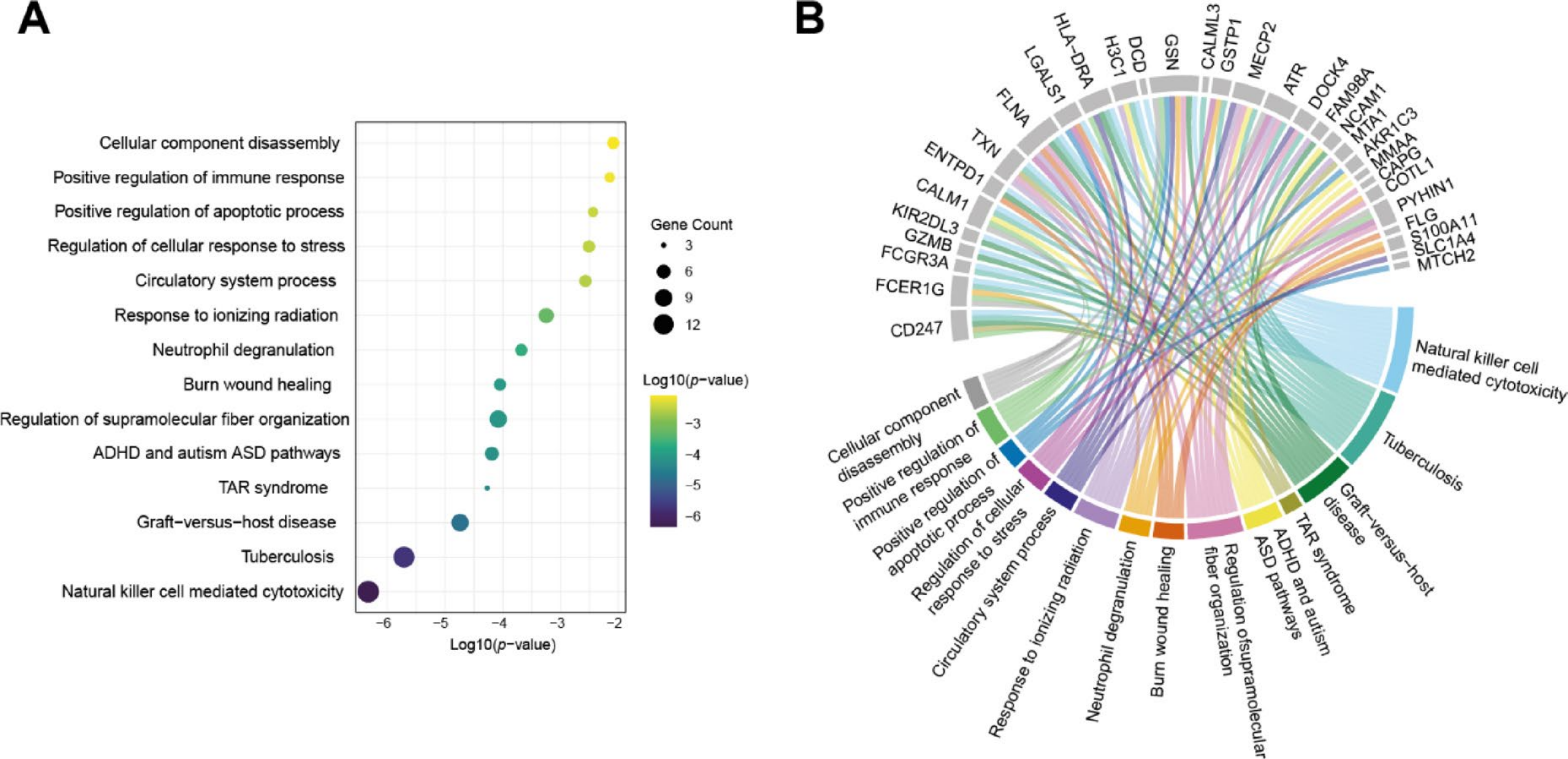
GO enrichment analysis of differentially expressed proteins. (**A**) Dot plot showing enriched GO Biological Processes for the 35 significant proteins, with gene counts and transformed *p*-values (log_10_). (**B**) Gene-concept network illustrating connections between significant proteins and GO terms.

Statistical analysis identified 35 differentially expressed proteins when comparing CD16− and CD16+ CIML NK cells. Functional enrichment analysis, conducted using Metascape (https://metascape.org)^23^ and Gene Ontology (GO) Biological Processes, along with KEGG pathway analysis, identified these proteins as being linked to key immune pathways. Notably, the analysis highlighted a significant overexpression of proteins associated with the “Natural Killer Cell-Mediated Cytotoxicity” pathway (Fig. 4A). Gene-concept network analysis of GO-enriched results (Fig. 4B) revealed that 14 of the differentially expressed proteins were directly linked to this pathway. Moreover, key genes such as CD247, FCER1G, CALM1, and HLA-DRA were associated with essential immune system functions. Enrichment terms for GO Biological Processes and related pathways are detailed in Supplementary Table 3.

**Fig. 5:**
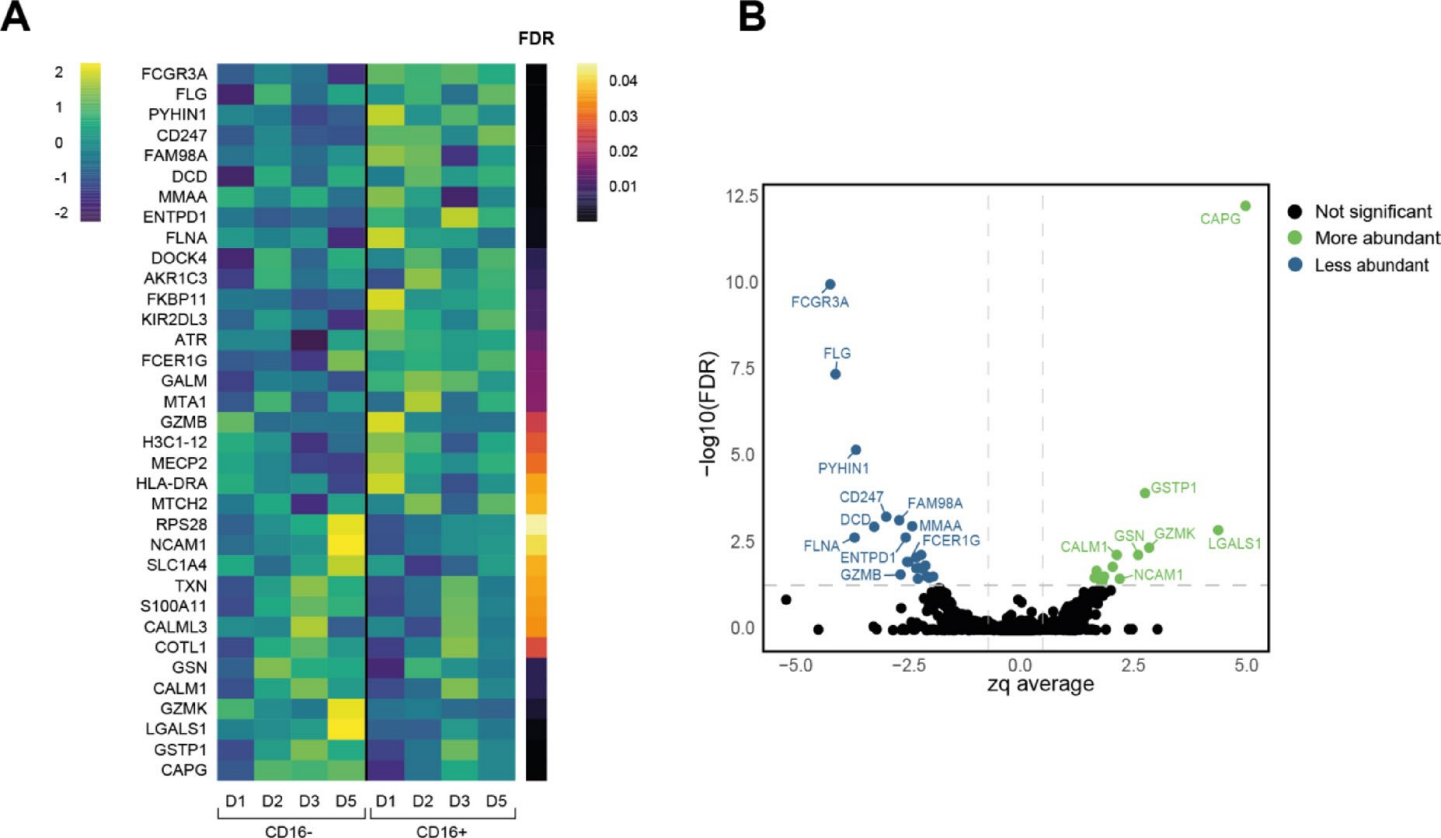
Proteomic analysis of sorted CD16− and CD16+ CIML NK cells. (**A**) Heatmap depicting the expression of the 35 most significantly different proteins (FDR < 0.05). (**B**) Volcano plot shows proteins with significant differential expression in CD16− versus CD16+ CIML NK cells.

To visualize the differences in protein expression between CD16− and CD16+ CIML NK cells, a heatmap (Fig. 5A) and a volcano plot (Fig. 5B) were generated. Among the 35 statistically significant changed proteins, 22 were less abundant in CD16− cells compared to CD16+ cells, while 13 were more abundant. Notably, proteins such as CAPG, GSTP1, CALM1, GSN, GZMK, LGALS1, and NCAM1 exhibited higher expression levels in CD16− cells. In contrast, proteins including FCGR3A, FLG, PYHIN1, CD247, DCD, FAM98A, MMA, FLNA, KIR2DL3, ENTPD1, GZMB, and FCER1G were more abundant in CD16+ cells.

## Discussion

Memory-like features of NK cells following cytokine stimulation were first described by Cooper et al.. in 2009 in mice^13^ and later by Romee et al.. in humans in 2012^14^. Since then, several studies have focused on characterizing CIML NK cells according to their phenotype, as well as their epigenetic and metabolic regulation^15,24^. This growing understanding of the biology of CIML NK cells has contributed to their incorporation into clinical trials, primarily targeting AML. CIML NK cell-based immunotherapy for AML has proven to be safe, well-tolerated, and capable of inducing clinical responses in patients with relapsed or refractory AML^20^, highlighting their potential as a promising strategy for AML treatment, whether as monotherapy or in combination with other therapies.

In this study, we conducted an in-depth characterization of CIML NK cells, discovering valuable insights that may be useful for the selection of the most effective CIML NK cell subsets for adoptive transfer in cancer patients. Notably, CIML NK cells are a heterogeneous population; we identified two major subsets based on CD16 expression, each exhibiting unique phenotypic and functional characteristics. Overnight stimulation with IL-12/15/18 results in a marked downregulation of CD16 expression. By Day 7 post-stimulation, there is a significant decrease in CD16+ CIML NK cells, which appears to be directly correlated with an increase in CD16− CIML NK cells.

The loss of CD16 expression has been suggested as a homeostasis mechanism triggered by activation-induced CD16 crosslinking^25^. Activation of NK cells induces cleavage of CD16 from the cell surface, a process mediated by matrix metalloproteinase MT6-MMP^26^ and ADAM-17^27^. Shedding of CD16 facilitates NK cell detachment from target cells, contributing to serial engagement of multiple targets and maintaining NK cell viability^8^. CD16 downregulation has been observed in NK cells in response to tumor stimulation^28^, cytokine stimulation^27,29^, upon activating receptor cross-linking^27^, and after vaccination^30^. In this context, and taking into account the lower expression of granzyme B in CD16− CIML NK cells, we should also consider that the decrease of CD16 in CIML NK cells may also be related to a degranulation process.

Our data shows that both subsets of CIML NK cells generated after IL-12/15/18 stimulation have higher expression of activation markers CD25 and CD69 compared to control NK cells (data not shown). By Day 7, no significant differences in these activation markers were observed between CD16+ and CD16− CIML NK cells, suggesting that the initial cytokine stimulation was equally effective for both subsets. Expansion of the CD16− subset was consistently observed in most donors following CIML induction.

Romee et al. demonstrated that both CD56bright and CD56dim peripheral blood NK cells can differentiate into CIML NK cells following stimulation with IL-12/15/18^14^. In our study, we found no correlation between the percentage of CD16 − CD56bright NK cells at Day 0 and the percentage of CD16− CIML NK cells at Day 7. Although we cannot discard the presence of CD56bright and CD56dim in the CD16− subset, our results support that the induction of a CD16− phenotype in CIML NK cells occurs independently of the initial percentage of the more immature CD56bright NK cell subset. These results suggest that the increase of CD16− CIML NK occurs after cytokine stimulation, not because of the preexisting proportion of CD56bright NK cells. Finally, while donor-dependent variability was observed, factors such as age and gender did not appear to influence the generation of a higher percentage of CD16− CIML NK cells.

Interestingly, by Day 7, CD16− and CD16+ CIML NK cell subsets displayed distinct phenotypic profiles. CD16− CIML NK cells expressed higher levels of activating receptors, including NKp30, NKp44, NKp46, NKp80, and DNAM-1, and lower levels of inhibitory receptors such as TIGIT, TIM-3, PD-1, and KIR2D. These findings suggest that CD16− CIML NK cells possess a more activated phenotype than CD16+ CIML NK cells. Additionally, NKG2A expression, a well-known marker of memory-like induction by cytokines^20,21,31^, was elevated in CD16− CIML NK cells, while NKG2C was more highly expressed in CD16+ CIML NK cells. NKG2C, along with CD57, is associated with CMV-induced adaptive NK cells^32,33^. In our study, we observed that CD16− CIML NK cells expressed lower levels of CD57 compared to CD16+ CIML NK cells (Supplementary Fig. 2), further highlighting the phenotypic differences between these subsets.

The phenotype of CD16− CIML NK cells suggests that these cells have a high functional capacity. Consistent with this, CD16− CIML NK cells exhibited greater degranulation in response to various target cells compared to CD16+ CIML NK cells. Considering our previous studies which demonstrated a positive correlation between degranulation capacity and TACTILE^21^, here we observed that TACTILE is expressed at higher levels in CD16− CIML NK cells compared to CD16+ cells. Moreover, its expression in CD16− CIML NK cells shows a significant positive correlation with their degranulation capacity, while no significant correlation was observed for the CD16+ subset (Supplementary Fig. 3).

It is important to note that, although the downregulation of CD16 is shown for K562 cells, no significant differences in CD16 expression were found when comparing CIML NK cells co-incubated with melanoma cell lines and control CIML NK cells. This suggests that the extent of CD16 downregulation may vary depending on the activation of NK cells against different target cells.

After characterizing CD16− and CD16+ CIML NK cells in terms of phenotype and degranulation capacity against various tumor cell lines, we conducted an in-depth proteomic analysis of these two subsets. While previous studies have also analyzed the proteomic profile of human NK cells^34^, various NK cell subsets^35^, NK cells isolated from different tissues^36^, NK cells during the interaction with tumor cells^37^, and IL-2 activated NK cells^38^, this work presents, to our knowledge, the first characterization of the proteomic content of in vitro-expanded CIML NK cells. Our analysis revealed that, as expected, a great percentage of the identified proteins were linked to the “Immune System” and “Innate Immune System” categories. The enrichment of NK cells from peripheral blood before IL-12/15/18 stimulation, and subsequent sorting of *in vitro-*expanded CD16− and CD16+ CIML NK cells ensured a highly purified population of NK cells for proteomic analysis.

The comparative analysis of proteomic profiles in CIML NK cells was performed using an FDR threshold of 0.05 ^39^ which is a widely used for controlling error rates^14^.

We identified 35 differentially expressed proteins between in vitro-expanded and sorted CD16− and CD16+ CIML NK cells. Notably, these proteins were significantly enriched in the KEGG pathway “Natural Killer Cell-Mediated Cytotoxicity” and corresponded to Gene Ontology terms such as “Immune System Process” and “Regulation of Biological Process”. Among these, 22 proteins were found in lower levels, and 13 were found in higher levels in CD16− cells compared to CD16+ cells.

As expected, FCGR3A (CD16/FcγRIII) was significantly lower in the CD16− subset^27^. Several studies indicate that CD16 shedding might enhance NK cell immune responses by facilitating detachment from target cells, which helps prolong NK cell survival and allows repeated targeting of multiple cells^8^. This paradox has led some researchers to propose that NK cell killing efficiency and kinase-driven tumor infiltration in immunotherapy may require careful interpretation when considering CD16 shedding^40^. The proteomic analysis also revealed a significant decrease of FCER1G (FcεRI gamma chain, FcεRIγ) and CD247 (CD3ζ) in CD16− CIML NK cells. Both FcεRIγ and CD247 are signaling adaptor molecules associated with CD16 and the natural cytotoxicity receptors (NCR) NKp30 and NKp46 ^41^. Therefore, these results further support that the loss of CD16 expression in CIML NK cells is linked with the decreased expression of these adaptor molecules found in the proteomic analysis despite the increased expression of NCR observed in CD16− CIML NK cells by flow cytometry. KIR2DL3 (CD158b) was also found to be significantly decreased in CD16− CIML NK cells. KIR2DL3, along with other KIR receptors, plays a crucial regulatory role in NK cell functionality^42^, and several clinical trials are currently investigating anti-KIR monoclonal antibodies to enhance NK cell-based therapies targeting lymphomas^43^, leukemia^44^, myeloma^45^, and solid tumors^46^. These findings highlight the potential significance of characterizing the KIR repertoire, particularly KIR2DL3 expression, in *in vitro-*expanded CIML NK cells to optimize their functional capacity for adoptive transfer.

Proteomic analysis identified 13 proteins, including NCAM1 and GZMK, that were significantly more abundant in the CD16− CIML NK cell subset compared to CD16+ CIML NK cells. The elevated expression of NCAM1 (CD56) in CIML NK cells has previously been observed using mass cytometry^20^. While the exact role of CD56 on CIML NK cells remains unclear, there is considerable evidence supporting the involvement of IL-15 in its up-regulation. In vitro culture with IL-15 has been shown to enhance CD56 expression in NK cells, accompanied by an increase in the expression of activating receptors and cytotoxic capacity^47^. The elevated levels of GZMK (Granzyme K) in CD16− CIML NK cells are also of interest. GZMK has been linked to innate-like lymphocytes^48^, and recent single-cell RNA sequencing meta-analyses have confirmed that GZMK expression is generally higher in CD16− NK cells compared to their CD16+ counterparts^49^.

Although further studies are needed to understand the role of CD16 in CIML NK cell function, the implementation of these findings in the clinic is necessary to improve the efficacy of NK cell transfer for the treatment of cancer patients.

In conclusion, CIML NK cells are a heterogeneous population; 7 days after cytokine stimulation, two distinct subsets can be differentiated according to CD16 expression. The CD16− subset demonstrates greater cytotoxicity, as evidenced by its enhanced degranulation capacity against various cell lines. This subset is further characterized by higher expression of NCR activating receptors and reduced expression of immune checkpoints such as PD-1, TIM-3, and TIGIT. Interestingly, TACTILE (CD96) expression is higher in CD16− CIML NK cells and positively correlates with their degranulation capacity, particularly in response to K562 cells, confirming our previous findings on CIML NK cells. The proteomic analysis further highlights the differences between these subsets, identifying unique immune biomarker phenotypes. These insights pave the way for selecting the most effective CIML NK cells to improve the efficacy of NK cell-based cancer immunotherapies.

## Materials and methods

### Sample processing and NK cell culture

NK cells were isolated from the buffy coats of nine healthy donors (Supplementary Table 1) and stimulated with cytokines to obtain CIML NK cells as previously described^21^. In brief, the enrichment of NK cells was performed using the RosetteSep™ Human NK Cell Enrichment Kit (STEMCELL Technologies, Vancouver, BC, Canada), followed by density gradient centrifugation with Lymphoprep™ (STEMCELL Technologies). The enriched NK cells (> 90% CD56+ CD3−) were then cultured at 37 °C in HyClone RPMI-1640 (Cytiva, Marlborough, MA, USA) supplemented with 1% sodium pyruvate, 1% GlutaMAX™ (Gibco™, ThermoFisher Scientific, Waltham, MA, USA), 1% penicillin-streptomycin (Lonza Ltd., Verviers, Belgium), and 10% human serum AB male (Biowest, Nuaillé, France). Cells were seeded at a density of 1 × 10^6^ cells/mL in 24-well plates and stimulated for approximately 16 h with 10 ng/mL recombinant human interleukin-12 (rhIL-12), 1 ng/mL rhIL-15 (PrepoTech, Rocky Hill, NJ, USA), and 50 ng/mL rhIL-18 (MBL International, Woburn, MA, USA). Following stimulation, cells were washed twice with phosphate-buffered saline (PBS) and re-seeded at a density of 1 × 10^6^ cells/mL in fresh 24-well plates containing 1 ng/mL rhIL-15. On Day 4, fresh culture medium with 1 ng/mL rhIL-15 was added.

This study was conducted in compliance with the ethical standards approved by the Ethics Committee of the University of Extremadura (Ref.: 118/2020), and informed consent was obtained from all donors, according to the principles of the Declaration of Helsinki.

### Cell lines

The human erythroleukemia cell line K562, along with the human melanoma cell lines MaMel45, MaMel51, MaMel56, IRNE, MEWO, ESTDAB-109, and ESTDAB-146 (obtained from the OISTER and ESTDAB projects), were utilized in this study. All cell lines were maintained in HyClone RPMI-1640 medium supplemented with 10% fetal bovine serum (Gibco™), 1% GlutaMAX™, and 1% penicillin-streptomycin. Melanoma cell lines were dissociated from culture flasks using CellStripper™ non-enzymatic cell dissociation solution (Corning^®^, Manassas, VA, USA).

### Flow cytometry analysis

The phenotypic and functional profiles of CIML NK cells were analyzed using multiparametric flow cytometry and a panel of commercially available antibodies (Supplementary Table 2). Prior to FACS analysis, the viability of cells was evaluated by trypan blue dye. The viability ranged from 95% to 100%. Extracellular staining was performed by incubating the cells with fluorochrome-conjugated antibodies at room temperature (RT) for 30 min. For intracellular staining, CIML NK cells were fixed and permeabilized using the IntraCell kit (Immunostep, Salamanca, Spain) following the manufacturer’s instructions. Intracellular antibodies were then added and incubated at RT for 30 min. After washing, cells were resuspended in PBS for flow cytometry analysis. Isotype controls and fluorescence-minus-one (FMO) controls were included to ensure proper gating (Supplementary Fig. 5).

Lymphocytes were gated based on forward scatter (FSC) and side scatter (SSC) characteristics. NK cells were identified as CD56+ CD3− cells within the lymphocyte gate. NK cells were further divided into two subsets based on CD16 and CD56 expression: CD16− CD56+ and CD16+ CD56+. Additional gates were set for each antibody to analyze the phenotypic profile of each NK cell subset. A full gating strategy is provided in Fig. 1A of the results section. Flow cytometry data were acquired using the MACSQuant Analyzer 10 (Miltenyi Biotec, Bergisch Gladbach, Germany) and analyzed with FlowLogic v.8.6 software (Inivai Technologies, Mentone, Victoria, Australia).

### NK cell degranulation assays

The functional capacity of CIML NK cells was evaluated by measuring their degranulation activity after seven days of culture. CIML NK cells (5 × 10^5^ cells) were co-cultured with target cells (K562, MaMel45, MaMel51, MaMel56, IRNE, MEWO, ESTDAB-109, and ESTDAB-146) at a 1:1 effector-to-target (E: T) ratio. To promote cell-to-cell contact, the co-cultures were centrifuged at 200*g* for 5 minutes. Degranulation was assessed by staining CIML NK cells with FITC-conjugated anti-CD107a (H4A3; BD Biosciences, San José, CA, USA) and FITC-conjugated anti-CD107b (H4B4; BD Biosciences). Cells were then treated with GolgiStop™ and GolgiPlug™ protein transport inhibitors (BD Biosciences) as per the manufacturer’s instructions and incubated at 37 °C with 5% CO_2_ and 95% humidity for 6 h. After incubation, CIML NK cells were stained with VioGreen^®^-conjugated anti-CD3, PE-Vio^®^770-conjugated anti-CD56, and VioBlue^®^-conjugated anti-CD16 monoclonal antibodies. Degranulation was analyzed by flow cytometry as described earlier.

### Sorting of CD16− CD56+ and CD16+ CD56+ NK cell subsets

Cells from four different donors were stained with fluorochrome-conjugated antibodies against CD3, CD56, and CD16 for 30 min at 4 °C, followed by two washes with PBS. Sample acquisition and initial gating were performed using FSC and SSC parameters. NK cell subsets (CD16− CD56+ and CD16+ CD56+) were sorted using the BD FACS Melody™ Cell Sorter (BD Biosciences) in purity mode. The sorter was equipped with a 488 nm blue direct diode laser (20 mW), a 405 nm violet direct diode laser (40 mW), and a 100 μm nozzle operating at 23 psi and 34 kHz. Before each sorting session, laser calibration was performed using BD™ CS&T RUO beads (BD Biosciences), and drop delay value was automatically calculated using BD™ FACS Accudrop RUO beads (BD Biosciences). Coulter Isoton II Diluent (Beckman Coulter Inc., Brea, CA, USA) was used as the sheath fluid during cell sorting.

The sorting rate ranged from 3,000 to 5,000 events per second, and collection continued until the entire sample was sorted. Data acquisition and analysis were performed using BD FACS Chorus Software (BD Biosciences).

### Proteomics

Proteomic analyses were performed on sorted NK cell subsets from four donors using a high-throughput multiplexed quantitative proteomics approach. Protein extracts from the CD16− CD56+ and CD16+ CD56+ subsets were subjected to trypsin digestion using Nanosep 30 K Omega filters (Pall Life Sciences, MA, USA), as previously described^50^. Protein digestion was carried out overnight at 37 °C with sequencing grade trypsin (Promega, Madison, WI, USA) at 1:40 (w/w) trypsin: protein ratio in digestion buffer (50 mM ammonium bicarbonate, pH 8.5). The resulting peptides were isobaric labeled with 8-plex iTRAQ (isobaric Tags for Relative and Absolute Quantitation) reagents (ABscience) following the manufacturer’s instructions. An aliquot of the labeled peptides was directly analyzed by LC-MS/MS while the remaining peptides were fractionated into five subgroups using the high pH reversed-phase peptide fractionation kit (Thermo Scientific), according to manufacturer’s protocol.

Liquid chromatography coupled to mass spectrometry (LC-MS/MS) analysis was done using an Evosep One HPLC (Evosep) coupled to an Orbitrap Eclipse Tribrid Mass Spectrometer (ThermoFisher Scientific) using an Endurance Evosep column 15 cm x 150 μm ID as analytical column (ThermoFisher Scientific) coupled to a stainless steel emitter of 30 μm ID. Peptides were eluted from Evotips and analyzed using the Evosep One pre-programmed gradient for 15 samples per day (SPD). Mass spectra were acquired in data-dependent manner, with an automatic switch between MS and MS/MS with a 2 s cycle time Top Speed method and 30 s dynamic exclusion. MS spectra were acquired in the Orbitrap analyzer using full ion-scan mode with a 375–1500 m/z range and 120,000 FT resolution. HCD fragmentation was performed at 36 normalized collision energy and MS/MS spectra were analyzed at 30,000 resolution in the Orbitrap. Two technical replicates per fractionated samples were analyzed.

For peptide identification, the raw LC-MS/MS data were searched using the SEQUEST HT algorithm implemented in Proteome Discoverer 2.5 (Thermo Scientific)^51^ against a UniProtKB^52^ database comprising human protein sequences (release November 2023) concatenated with decoy sequences generated using DecoyPyrat^53^. The false discovery rate (FDR) was calculated using the corrected Xcorr score (cXcorr)^54^ and the target/decoy competition strategy applying the picked FDR method at the peptide level^55^, with an additional filter for precursor mass tolerance of 15 ppm^50^. A 1% FDR was employed as the criterion for peptide identification. The quantitative information was extracted from the iTRAQ reporter intensity in the raw LC-MS/MS data and the quantification of protein abundances changes across lysates was carried out on the basis of the WSPP model^56^ and the Systems Biology Triangle algorithm^57^ using iSanXoT software package (version 2.1.0) ^58^.

### Statistical analysis and graphical representation

Flow cytometry data normality was assessed using the Shapiro-Wilk test before statistical analysis. For multiple group comparisons, the non-parametric Friedman test was used, followed by pairwise comparisons with the Durbin-Conover test. Paired sample comparisons were performed using the non-parametric Wilcoxon signed-rank test. All statistical analyses were conducted using the open-source software Jamovi v2.5.5 (Sydney, Australia).

In the proteomic analysis, the standardized variable Zq was defined as the mean-corrected log_2_ ratio between CD16− CD56+ and CD16+ CD56+ subsets, expressed in units of standard deviation at the protein level. The Limma package^58^ was used to ascertain statistical significance by means of *p*-values. Multiple test correction was applied using the Benjamini-Hochberg False Discovery Rate (FDR), with a threshold of FDR < 0.05 indicating statistically significant protein abundance changes. Proteomics data have been submitted to the ProteomeXchange Consortium and are available through the PRIDE partner repository^59^ (Dataset identifier: PXD059190).

Pathway and biological process enrichment analyses were performed using the Metascape platform (https://metascape.org/) ^23^. Differentially expressed proteins were further analyzed through databases including KEGG Pathway^60,61^, GO Biological Processes, Reactome Gene Sets, Canonical Pathways, CORUM, and WikiPathways. *P*-values for these analyses were calculated based on the cumulative hypergeometric distribution.

Flow cytometry data were visualized using GraphPad Prism v10.2.0 (San Diego, CA, USA), while proteomics data, including enrichment dot plots, gene-concept networks, heatmaps, and volcano plots, were analyzed and visualized using RStudio v 2024.09.1 + 394 and R v4.4.2.

## Supplementary Information

Below is the link to the electronic supplementary material.

Supplementary Material 1

## Data Availability

The raw data supporting the conclusions of this article will be made available by the authors, without undue reservation. The mass spectrometry proteomics data have been deposited to the ProteomeXchange Consortium with the dataset identifier PXD059190 (https://www.ebi.ac.uk/pride/archive/projects/PXD059190).
