# Supplementary material for: Phenotypic, proteomic, and functional analyses of cytokine-induced memory-like NK cells show two distinct subsets based on CD16 expression
Source: Sci Rep. 2025 Oct 23;15:37053. doi: 10.1038/s41598-025-20947-1 (PMC12549889; doi:10.1038/s41598-025-20947-1)
Supplement: Supplementary file 1 — Supplementary Material 1 [file 41598_2025_20947_MOESM1_ESM.docx]

***SUPPLEMENTARY MATERIAL***

**Phenotypic, proteomic, and functional analyses of cytokine-induced memory-like NK cells show two distinct subsets based on CD16 expression**

Sofía Carreira-Santos^1^, Marina González-Sánchez^1^, Nelson López-Sejas^1^, Fakhri Hassouneh^2^, Lauro González-Fernández^3^, Inmaculada Jorge^4^, Esther Durán^5^, Alejandra Pera^2,6^, Jesús Vázquez^4^, Rafael Solana^2,6,7^*, Raquel Tarazona^1,8^*, Javier G. Casado^1,8,9*^

^1^ Immunology Unit, Department of Physiology, Universidad de Extremadura, Cáceres, Spain; ^2^ Department of Cell Biology, Physiology and Immunology, Universidad de Córdoba, Córdoba, Spain; ^3^ Departamento de Bioquímica y Biología Molecular y Genética, Grupo de Investigación Señalización Intracelular y Tecnología de la Reproducción (SINTREP), Instituto de Investigación INBIO G+C, Facultad de Veterinaria, Universidad de Extremadura, Cáceres, Spain; ^4^ Cardiovascular Proteomics Laboratory, Centro Nacional de Investigaciones Cardiovasculares Carlos III (CNIC), Madrid, Spain; Centro de Investigación Biomédica en Red, Enfermedades Cardiovasculares (CIBERCV), Madrid, Spain; ^5^ Anatomy and Comparative Pathological Anatomy Unit, Department of Animal Medicine, Faculty of Veterinary Medicine, Universidad de Extremadura, Cáceres, Spain; ^6^ Immunology and Allergy Group (GC01), Maimonides Biomedical Research Institute of Córdoba (IMIBIC), Córdoba, Spain^;^ ^7^ Immunology and Allergy Service, Reina Sofia University Hospital, Cordoba, Spain; ^8^ Institute of Molecular Pathology Biomarkers, Universidad de Extremadura, Cáceres, Spain; ^9^ RICORS-TERAV Network, Instituto de Salud Carlos III (ISCIII), Madrid, Spain.

*** Corresponding Authors:**Raquel Tarazona rtarazon@unex.es

Rafael Solana rsolana@uco.es

Javier G Casado jgarcas@unex.es

**Supplementary Figures and Tables**

**Supplementary Table 1. Demographics and proteomic analysis details of healthy donors included in the study.**

| Donor ID | Age (years) | Sex |
| --- | --- | --- |
| D1 | 28 | Female |
| D3 | 34 | Male |
| D3 | 22 | Male |
| D4 | 57 | Female |
| D5 | 34 | Male |
| D6 | 58 | Male |
| D7 | 24 | Female |
| D8 | 55 | Male |
| D9 | 53 | Female |

**Supplementary Table 2. Antibody panel used for extracellular and intracellular staining in the phenotypic characterization of CIML NK cells.**

| Extracellular staining | | | |
| --- | --- | --- | --- |
| Antibody | **Fluorochrome** | **Provider** | **Clone** |
| CD16 | VioBlue^®^ | Miltenyi Biotec | REA423 |
| CD3 | VioGreen^®^ | Miltenyi Biotec | REA613 |
| CD25 | FITC | Invitrogen | CD25-3G10 |
| NKG2A | PE-REA | Miltenyi Biotec | REA110 |
| CD8 | PerCP-Cy5.5 | BD Biosciences | SK1 |
| CD56 | PE-Vio^®^770 | Miltenyi Biotec | REA196 |
| NKG2D | APC | BD Biosciences | 1D11 |
| CD69 | APC-Vio^®^770 | Miltenyi Biotec | REA824 |
| NKG2C | FITC | Miltenyi Biotec | REA205 |
| NKp80 | APC-Vio^®^770 | Miltenyi Biotec | REA845 |
| DNAM-1 | FITC | BD Biosciences | DX11 |
| TIGIT | PE | Invitrogen | MBSA43 |
| TACTILE | APC-REA | Miltenyi Biotec | REA195 |
| Lag-3 | BV421 | BD Biosciences | T47-530 |
| TIM-3 | BB515 | BD Biosciences | 7D3 |
| PD-1 | APC | BD Biosciences | MIH4 |
| CD16 | APC-Vio^®^770 | Miltenyi Biotec | REA423 |
| NKp44 | Vio®Bright-FITC | Miltenyi Biotec | 2.29 |
| NKp46 | PE | BD Biosciences | 9-E2 |
| NKp30 | APC | Miltenyi Biotec | AF29-4D12 |
| KIR2D | PE-REA | Miltenyi Biotec | REA1042 |
| Intracellular staining | | | |
| Antibody | **Fluorochrome** | **Provider** | **Clone** |
| Perforin | VioBlue^®^ | Miltenyi Biotec | 𝛿G9 |
| Granulysin | AF488 | BD Biosciences | RB1 |
| Granzyme B | AF647 | BD Biosciences | GB11 |

**Supplementary Table 3. Pathway and Biological Process enrichment analysis conducted via Metascape.** The enrichment analysis included KEGG Pathways, Gene Ontology (GO) Biological Processes, Reactome Gene Sets, Canonical Pathways, CORUM, and WikiPathways.

| Pathway/Process | Category | Term | Gene Count | Log10(*p*-value) | Enriched Genes |
| --- | --- | --- | --- | --- | --- |
| Natural killer cell mediated cytotoxicity | KEGG Pathway | hsa04650 | 14 | -6.325759349 | CD247, FCER1G, FCGR3A, GZMB, KIR2DL3, CALM1, ENTPD1, TXN, FLNA, LGALS1, HLA-DRA, H3C1, DCD, GSN |
| Tuberculosis | KEGG Pathway | hsa05152 | 13 | -5.708988129 | CALM1, CALML3, FCER1G, FCGR3A, HLA-DRA, GSTP1, TXN, FLNA, MECP2, ATR, CD247, H3C1, DOCK4 |
| Graft-versus-host disease | KEGG Pathway | hsa05332 | 9 | -4.743880957 | GZMB, HLA-DRA, KIR2DL3, CD247, FLNA, GSN, FAM98A, ENTPD1, NCAM1 |
| TAR syndrome | WikiPathways | WP5362 | 3 | -4.273511729 | CD247, TXN, MTA1 |
| ADHD and autism ASD pathways | WikiPathways | WP5420 | 6 | -4.194371695 | CALM1, MECP2, NCAM1, AKR1C3, MMAA, H3C1 |
| Regulation of supramolecular fiber organization | GO Biological Processes | GO:1902903 | 9 | -4.081708958 | CAPG, FLNA, GSN, MECP2, COTL1, ATR, TXN, DOCK4, PYHIN1 |
| Burn wound healing | WikiPathways | WP5055 | 5 | -4.047075641 | FLG, LGALS1, S100A11, ENTPD1, FLNA |
| Neutrophil degranulation | Reactome Gene Sets | R-HSA-6798695 | 5 | -3.67563531 | FCER1G, GSN, GSTP1, S100A11, COTL1 |
| Response to ionizing radiation | GO Biological Processes | GO:0010212 | 7 | -3.247949794 | ATR, MECP2, MTA1, TXN, FLNA, FAM98A, H3C1 |
| Circulatory system process | GO Biological Processes | GO:0003013 | 5 | -2.566079188 | GSN, MECP2, SLC1A4, DOCK4, HLA-DRA |
| Regulation of cellular response to stress | GO Biological Processes | GO:0080135 | 5 | -2.514851658 | ATR, FLNA, GSTP1, PYHIN1, LGALS1 |
| Positive regulation of apoptotic process | GO Biological Processes | GO:0043065 | 4 | -2.439994184 | GSN, LGALS1, AKR1C3, MTCH2 |
| Positive regulation of immune response | GO Biological Processes | GO:0050778 | 4 | -2.146536329 | CD247, FCER1G, HLA-DRA, PYHIN1 |
| Cellular component disassembly | GO Biological Processes | GO:0022411 | 5 | -2.08678638 | ATR, CALM1, GSN, FCER1G, PYHIN1 |


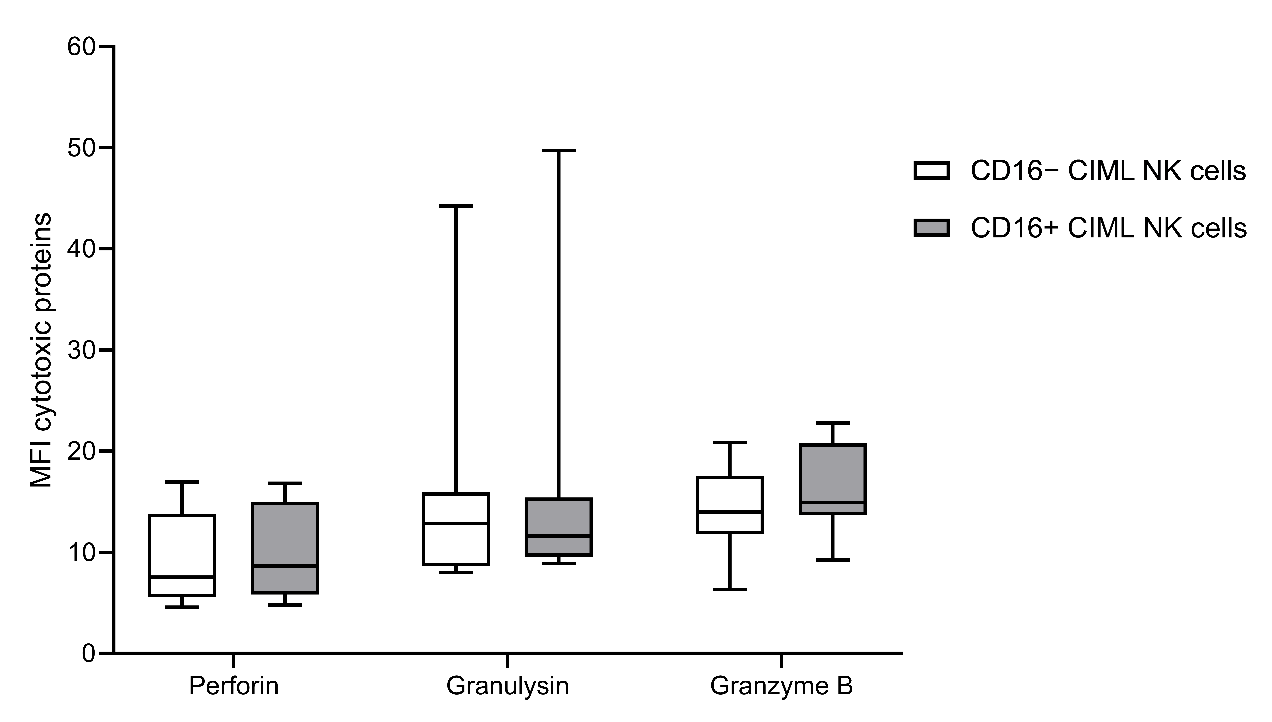


**Supplementary Figure 1. Median Fluorescent Intensity (MFI) of cytotoxic proteins in CD16− and CD16+ and CIML NK cells**. Statistical significance was determined using the Wilcoxon signed-rank test.


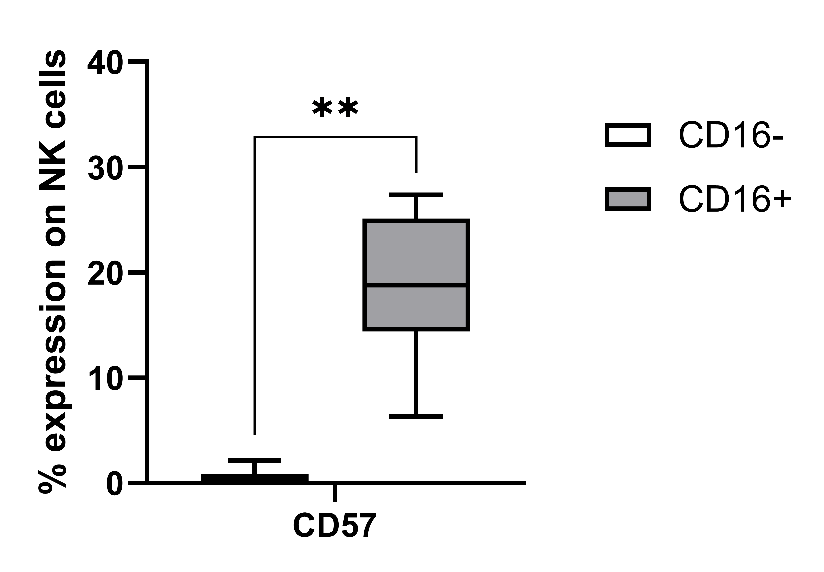


**Supplementary Figure 2. CD57 expression on CD16− and CD16+ CIML NK cells after 7 days of culture.** CD16**−** CIML NK cells express significantly less CD57 than the CD16+ subset. Statistical significance was determined using the Wilcoxon signed-rank test, *p* = 0.004.


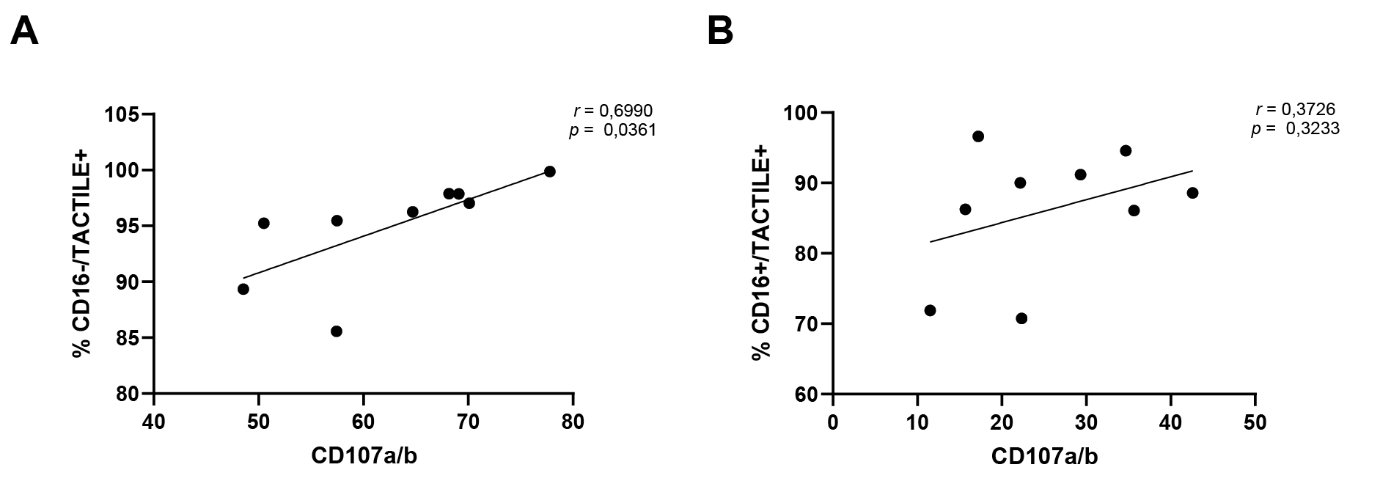


**Supplementary Figure 3. Correlation analysis of TACTILE and CD16 co-expression with NK cell degranulation in CD16 CIML NK cell subsets. (A)** TACTILE expression on CD16− CIML NK cells shows a significant positive correlation with degranulation, as measured by CD107a/b expression. **(B)** In contrast, no significant correlation was observed for the CD16+ subset.

**
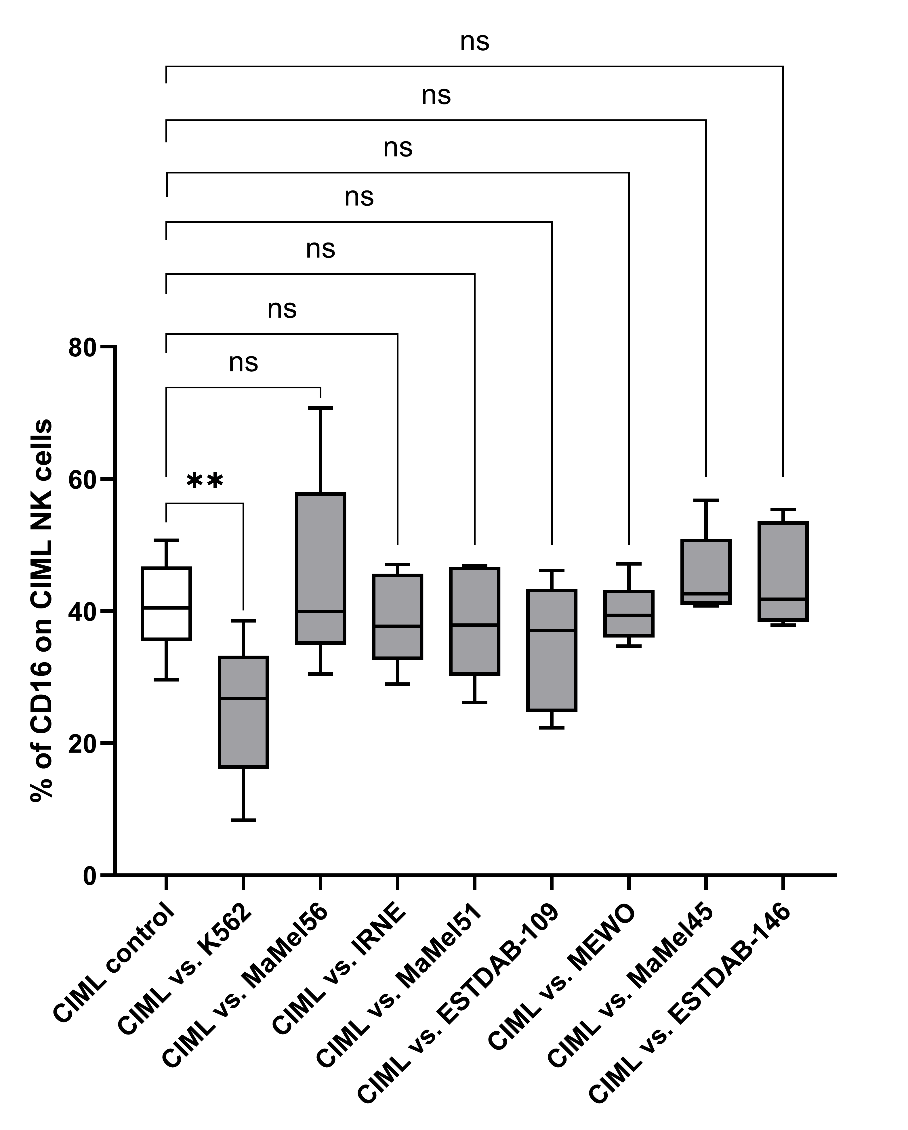
**

**Supplementary Figure 4. CD16 expression on CIML NK cells after co-incubation with K562 and melanoma cell lines.** CIML NK cells experienced a significant downregulation of CD16 expression when co-incubated with K562 target cell compared to control CIML NK cells (spontaneous degranulation). Statistical significance was determined using the Wilcoxon signed-rank test, *p* = 0.004, ns=not significant.

**
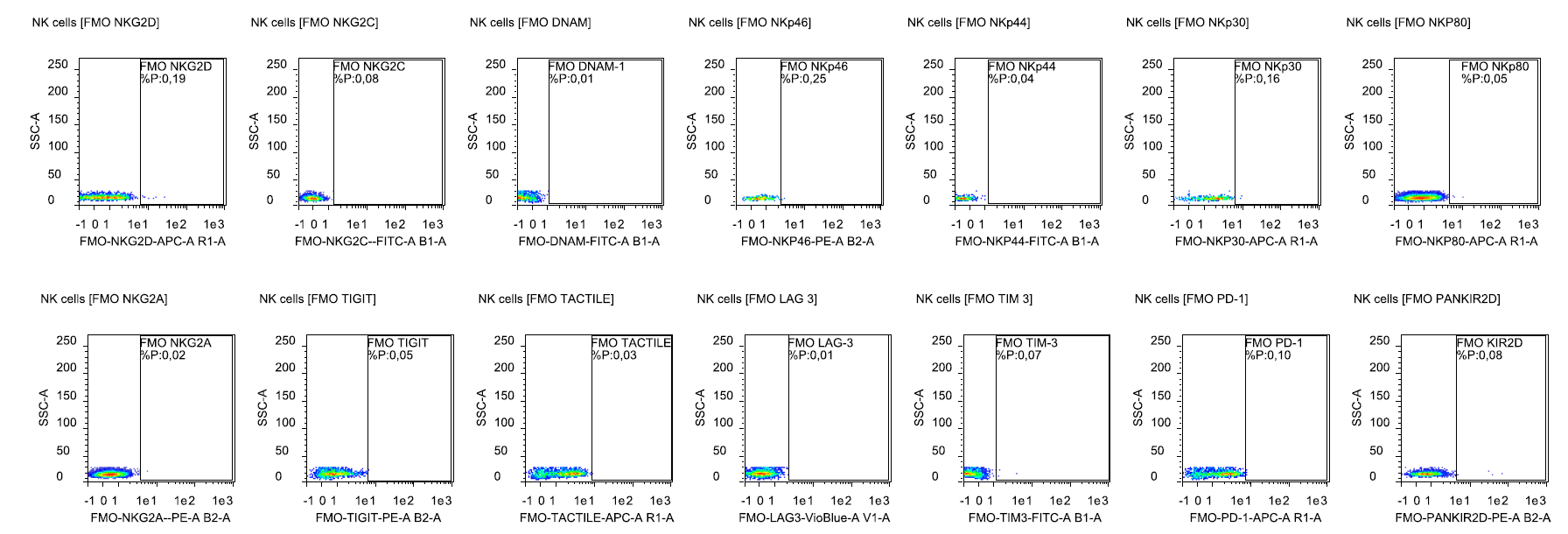
**

**Supplementary Figure 5.** Fluorescence minus one (FMO) analysis of NK cells. NK cells were defined as CD3− CD56+ cells within the lymphocte gate. FMO controls were used to establish negative thresholds for activating and inhibitory receptors.
